# Groundwater extraction-induced seismicity around Delhi region, India

**DOI:** 10.1038/s41598-021-89527-3

**Published:** 2021-05-12

**Authors:** Deepak K. Tiwari, Birendra Jha, Bhaskar Kundu, Vineet K. Gahalaut, Naresh K. Vissa

**Affiliations:** 1 Department of Earth and Atmospheric Sciences, NIT Rourkela, Rourkela, 769008 India; 2 Department of Chemical Engineering and Materials Science, University of Southern California, Los Angeles, CA 90007-1211 USA; 3 CSIR-National Geophysical Research Institute, Uppal Road, Hyderabad, 500007 India

**Keywords:** Geophysics, Hydrogeology, Seismology, Tectonics

## Abstract

The non-tectonic deformation, either of natural or anthropogenic origin, may influence the earthquake occurrence process and seismicity rate along the plate-boundary or ‘stable’ plate-interiors domains. The low magnitude but moderate seismicity rate of Delhi region on the stable plate-interiors domains of India, exhibits significant variation both in short-term at annual seasonal scale and in long-term at decadal scale. It correlates with the anthropogenic groundwater pumping for the extensive irrigation, urban activities, and seasonally controlled hydrological loading cycle of Indo-Ganga Basin hosted freshwater aquifers. Our coupled hydro-mechanical simulation and poro-mechanical analysis of basement fault stability suggest that the combined aquifer contraction and basement rock expansion act together to modulate the effective stress regime and anthropogenic seismicity on the basement faults in Delhi region.

## Introduction

Human-induced earthquakes are increasingly becoming a matter of focal point of socio-political and scientific concern in the past few decades^1–7^). Globally increasing reports of earthquakes around oilfields (Cavone, Italy^8^), geothermal fields (Basel, Switzerland^9^), groundwater pumping sites (Lorca, Spain^1^), gas storage sites (Castor, Spain^10^), water injection sites (KTB Germany^11^; Paradox Valley USA^12,13^), hydraulic fracturing^14^, and carbon sequestration sites^15^ over the last decades have raised an alarm in the energy industry and geoscience community. Worldwide several anthropogenic activities have been documented to link with seismicity and based on that an updated database, *HiQuake* has been created for documenting Human-induced earthquake^4,5^.

Such anthropogenic activities modify the subsurface stress regime of the critically stressed fault system, inducing earthquakes, altering the seismicity rate in the surrounding region, and making the region more susceptible to dynamic triggering by remote distant earthquakes^4,5^. Unlike, the natural tectonic earthquakes, the Human-induced earthquakes are frequently associated with blind basement faults making it challenging for geoscientists to assess associated seismic hazard. Therefore, seismic hazard assessment and mitigation of the Human-induced earthquakes require identification of seismogenic fault system and characterization of the state-of-stress in the subsurface.

**Figure 1:**
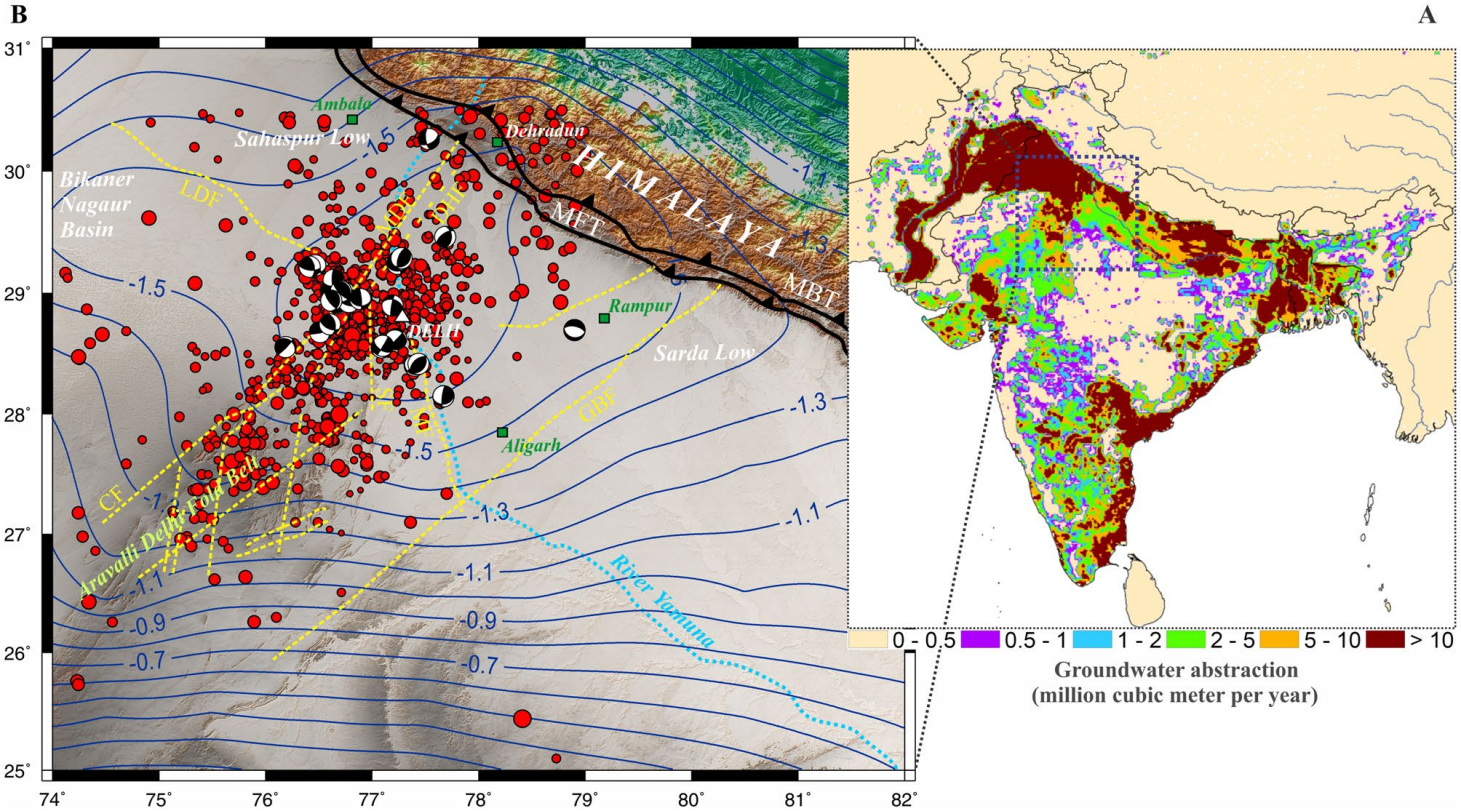
Groundwater extraction from the Indo-Ganga plain, Delhi surrounding region and Aravalli Delhi fold belt associated seismicity. **(A)** The groundwater pumping rate of the Indian subcontinent derives from International Groundwater Resources Assessment Center (IGRAC) and after that it was downscaled to grid scale using the deficit of surface water availability over the total water demand as a proxy. Note groundwater abstraction rate is highest in the Indo-Ganga plain and Delhi surrounding region. **(B)** Rate of groundwater depletion of the region (marked by white box in **A**) derived from GRACE and GLDAS (contours in cm/year). Note the Aravalli Delhi fold belt associated seismicity lies in the region of maximum groundwater depletion (~ 1.6 cm/year). Different basement faults are marked by dashed lines^38^. *MDF* Mahendragarh Dehradun Fault, *CF* Chahapoli Fault, *SF* Sohna Fault, *MF* Mathura Fault, *GBF* Great Boundary Fault, *DHF* Delhi Haridwar Fault, *MBT* Main Boundary Thrust, *MFT* Main Frontal Thrust. This figure was generated using Generic Mapping Tools (version 5.2.1; URL: http//gmt.soest.hawaii.edu/).

In this letter, we have strengthened *HiQuake* database^4,5^, by presenting a compelling scenario of groundwater extraction-induced seismicity around the Delhi region of Northern India (Fig. 1). Using continuous modern seismicity records around Delhi surrounding region, combined Gravity Recovery and Climate Experiment (GRACE) data, hydrological and coupled hydromechanical simulation and associated poromechanical analysis of fault stability, we suggest that the seismicity around the Delhi region, which is linked with the Aravalli Delhi fold belt, is possibly influenced by the non-tectonic deformation process including anthropogenic groundwater pumping for the extensive irrigation and urban activities, along with seasonally controlled hydrological loading cycle in the Northern India.

## Aravalli Delhi fold belt associated seismicity

The seismicity in the Aravalli Delhi fold belt region is possibly linked with the paleo-structure which experienced rifting, stretching and collision in geological past, about 160–200 Ma^16^. Although entire Aravalli Delhi fold belt has experienced earthquakes, the Delhi region appears to be more active seismically and it has witnessed several moderate and strong historical earthquakes, e.g., in 1720, 1831, 1956, 1960^17,18^. Besides these, several small magnitude earthquakes have occurred in the region which has been recorded by a dense network operating in the region, particularly since the beginning of this century. Majority of these earthquakes occur in the upper 25 km of the crust and they exhibit significant variation in earthquake focal mechanisms. However, majority of them involve reverse motion along with strike-slip motion on steep planes^17^ (Fig. 1B). Despite variation in the focal mechanisms ^18–21^, the resulting stress direction inverted from the available focal mechanism solutions is largely consistent with a stress regime in which the maximum principal stress is in the NNE-SSW direction with a moderate plunge^17^ which is similar to that in the neighboring Garhwal Kumaun region^22^.

## Results

### Seasonal modulation of seismicity in Aravalli Delhi folds belt region

Previous studies have noted seismicity modulation at annual periods in ‘stable’ plate interiors and proposed that annual stress change of a few kPa are capable of modulating seismicity in seismically active but low-strain region^23,24^. Recently, Yadav et al.^17^ has analyzed the current and historical seismicity of the Aravalli Delhi fold belt and also noted that the seismicity in the Delhi region has some seasonal variation. In order to make these observations more robust, we generated an aftershock-depleted relocated earthquake catalogue from 2000 to 2020, controlled by National Center of Seismology, Delhi, using the declustering approach^25^. To establish the completeness threshold of the catalog (Mc), we analyzed the Gutenberg-Richter relation of the raw catalog of the region during the study period (2000–2020) using a maximum likelihood approach^26^. We find that the lower magnitude threshold (Mc = 2.5) in the catalogue remains stable over time during the observation period (Fig.S1).

**Figure 2:**
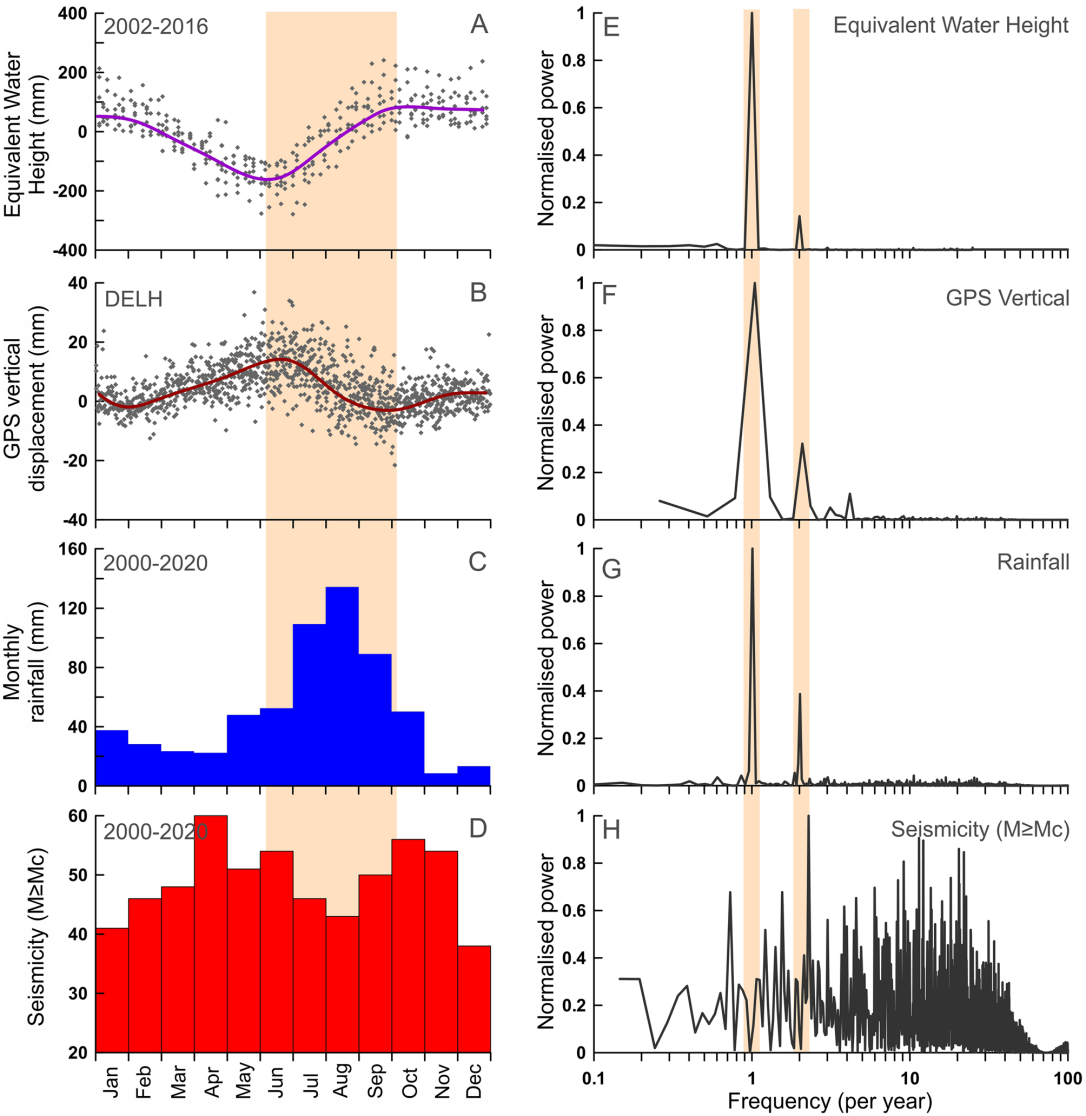
Correlation between Equivalent Water Height, GPS derived vertical displacement, rainfall, and seismicity frequency from the Aravalli Delhi fold belt. **(A–D)** represents monthly stacked time series of equivalent water height, GPS vertical displacement (station DELH), rainfall derived from TRMM, and monthly seismicity (M $\ge Mc$). Orange strip represent the hydrological loading period. **(E–H)** represents Power spectrum analysis of equivalent water height, GPS displacement, rainfall and seismicity catalogue from Aravalli Delhi fold belt region. Note that equivalent water height, GPS displacement and rainfall shows strong annual and weak semi-annual periodicity. Note seismicity level is relatively lower during time of seasonal hydrological loading period (light red shading in **A–D**). This figure was generated using Grapher graphical application (version 8.7.844 URL: https://www.goldensoftware.com/products/grapher).

Visual inspection of the time series of GRACE-derived equivalent water height (EWH), GPS derived vertical displacement, regional rainfall, and seismicity (of M ≥ Mc), clearly suggest that the earthquake occurrences have good correlation with the timing of the seasonal hydrological loading cycle in the Delhi surrounding region (Fig. 2A–D). Further, to make this visual inspection statistically significant, we have computed periodicity using the Power spectrum analysis of various physical parameters (Fig. 2E–H). From this analysis it has appeared that equivalent water height (EWH), GPS derived vertical displacement and regional rainfall exhibit strong annual periodicity along with relatively weak semi-annual periodicity (Fig. 2E–H). Interestingly, seismicity of the Delhi region clearly exhibits strong semi-annual periodicity. Moreover, during seasonal loading period (June–September, i.e., during monsoon), the seismicity is lowest whereas the seismicity level is relatively high during the unloading period. Therefore, we propose that the precipitated water load during monsoon period and seasonal recharge of the regional aquifer stabilize the causative faults in the basements, but the same faults are destabilized during seasonal unloading period. This seasonal modulation of seismicity prompted us to explore whether the increased extraction of the groundwater (i.e., long-term decadal unloading) also influence the seismicity in the region.

### Groundwater pumping and long-term seismicity modulation in Aravalli Delhi fold belt region

Recent hydrological modeling, in situ observation and satellite-based Earth observations have indicated alarming rates of groundwater pumping (extraction), leading to rapid depletion of freshwater aquifers around the world, such as north-western India, the Northern China Plain, the central USA, California, Tigris River plain and western Iran region including some parts of Middle East^27^. In fact, the rate of groundwater extraction is quite alarming for the Indo-Gangetic Basin associated alluvium freshwater aquifer in the north-western India^28^. The aquifer system in the Indo-Ganga plains is formed by the sediments eroded from the Himalayan Mountain which are subsequently redistributed by the Indus, Ganges and Brahmaputra river system, resulting in alluvium freshwater aquifer on the fertile plains across Pakistan, northern India and Bangladesh^29^ (Fig. 1). Specifically, the Delhi and surrounding region is covered by this alluvium which is occasionally traversed by the linearly elongated quartzite ridges of Proterozoic age, which act as a basement rock of the area^30,31^. The thickness of the alluvium has been reported up to 300 m in the immediate vicinity of the Delhi region but increases in the north to about 6 km^32^.

**Figure 3:**
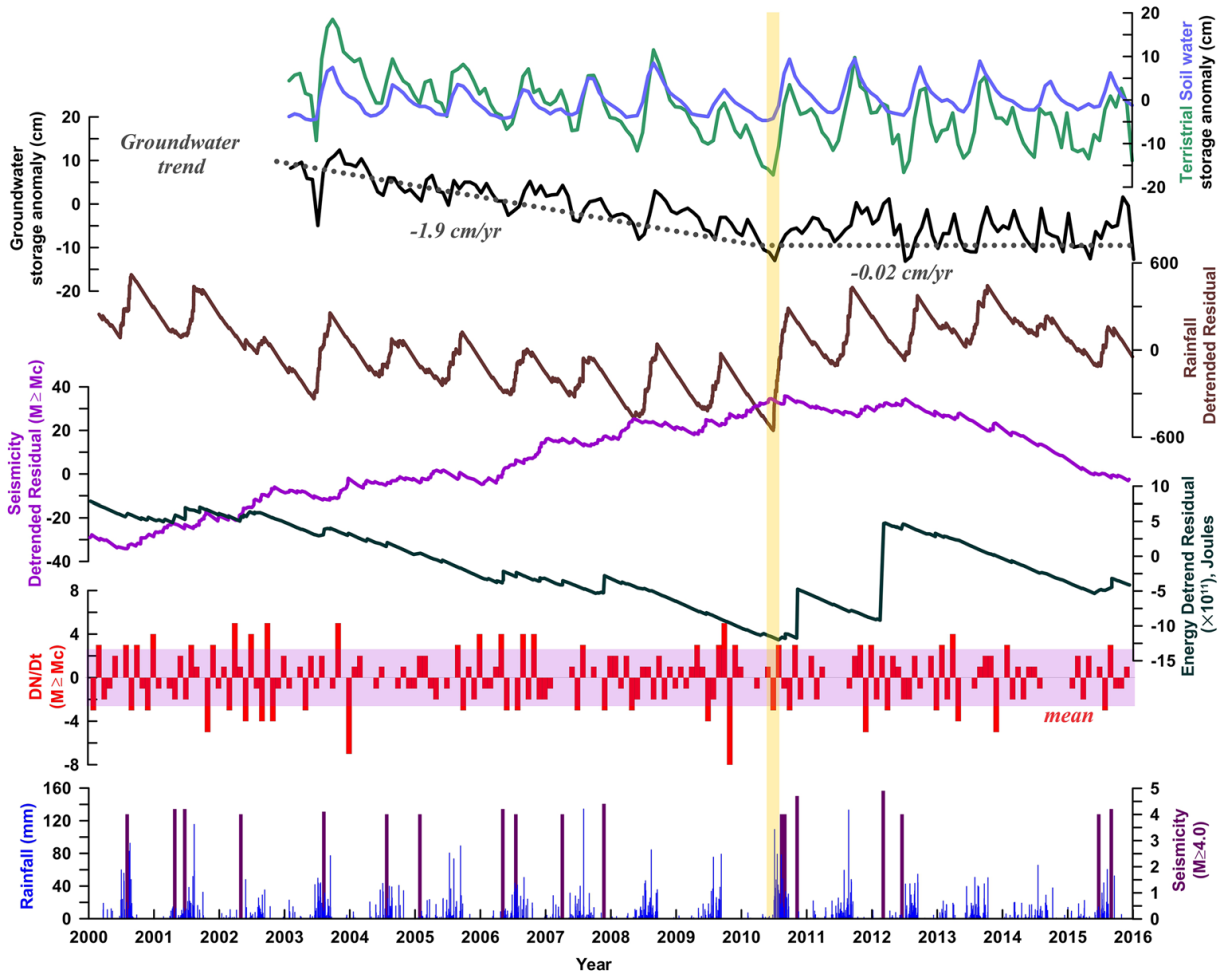
Time series representation for Terrestrial, Snow water, and Groundwater storage anomaly, which are derived from the GRACE and GLDAS observations in the study region. Note the steady declining trend in groundwater (~ 1.9 cm/year) from 2002 to 2009 which becomes stable afterwards. The transition is marked by the yellow strip. The cumulative change in Aravalli Delhi fold belt associated seismicity, seismic energy, seismicity rate (denoted by Dn/Dt) and regional rainfall also presented in the lower four panes. Note the change in the slope of the detrended cumulative seismicity coincides with the trend change in the groundwater storage anomaly and detrended cumulative regional rainfall (i.e., marked by the yellow strip). This figure was generated using Grapher graphical application (version 8.7.844 URL: https://www.goldensoftware.com/products/grapher).

In order to quantify groundwater extraction rate in the north-western India and Delhi surrounding region, we have explored 156 months of Gravity Recovery and Earth Climate Experiment (GRACE) data and Global Land Data Assimilation System (GLDAS), from January 2003 to December 2015 to quantify the rate of decrease in total water storage and groundwater changes (described in Methods). From this analysis, we estimate the rate of groundwater storage change as 1.6 ± 0.6 cm/year during 2002 to 2015 which is alarming in the Delhi surrounding region of the Indo-Ganga Basin (Fig. 3). This is consistent with the earlier estimates from the period of 2003–2009^28,33^. Wada et al.^34^, developed a global hydrological and water resource model to estimate groundwater extraction, which is also consistent with our analysis of satellite-based observations (Fig. 1A). Further, another important aspect to note is that there is substantial decline in groundwater storage change (1.9 ± 0.6 cm/year) from the period of 2003–2009. However, the groundwater storage change remained relatively stable (0.02 ± 0.5 cm/year) during 2010–2015 (Fig. 3), particularly in the Delhi region. The groundwater storage change appears to be linked to the temporal change in regional rainfall and associated recharge of the Delhi surrounding region (i.e., represented by the detrended cumulative change in daily rainfall rates, Fig. 3, Fig.S2).

Interestingly, such a trend change in groundwater storage is also correlated with the cumulative change in the seismicity rates in the Aravalli Delhi fold belt during the same period (Fig. 3). Figure 3 shows time series of terrestrial, snow water, and groundwater storage anomaly, derived from the GRACE and GLDAS observation, along with the monthly seismicity rate change (represented by dN/dt) for declustered catalog (M ≥ Mc), and detrend-cumulative change in the corresponding seismicity rates. The time series analysis suggests that the monthly seismicity rate change and the cumulative change of Aravali Delhi fold belt associated seismicity rates are well correlated with the trend change in groundwater storage. In order to make our observations more robust (i.e., presented in Figs. 2 and 3), we have presented cross-correlation analysis among various physical parameters in the supporting documents (Fig. S3). From this analysis it is confirmed that the phase relationship between hydrological loading and precipitation is complex, as much of the water mass is temporarily stored in surface reservoirs and groundwater table thus delaying (~ 2–3 months) its transport out of the system. Further, GRACE-derived EWH and regional rainfall exhibit strong lag-correlation with the occurrence of seismicity. However, we do not find any strong correlation between rainfall/groundwater storage anomalies and the released energy associated with the seismicity. Therefore, it appears that monthly seismicity rate change is significantly higher during the decline in groundwater storage change in 2003–2010, as compared to the later period. This is also evident from the change in the slope of the detrend-cumulative seismicity rate. To check the stability of the results, we even analyzed the raw earthquake catalogue (i.e., without applying declustering approach, and without the consideration of Mc) and found no significant difference in the results. Therefore, it implies that the seismicity change around the Aravalli Delhi fold belt is possibly influenced by the anthropogenic groundwater pumping.

### Coupled poromechanical model and fault stability due to groundwater extraction

In order to probe the anthropogenic groundwater pumping-induced seismicity modulation, we use a novel approach of coupled poromechanical analysis considering groundwater withdrawal and subsequent unloading of the crust over the period of interest and assessed its influence on the basement faults. We developed a quasi-2-D coupled flow-geomechanical model for the Delhi region to investigate the basement fault stability, change in stress regime due to groundwater extraction, and associated crustal unloading process over the period of two decades (see supplementary material and method).

The higher rates of groundwater extraction from the alluvium unconfined aquifer of the Delhi and surrounding region during 1985–2005 has led to an estimated 20–25 m drop in the water table^29,31^. Estimating the corresponding subsidence associated with the groundwater extraction is more difficult due to the uncertainties surrounding the withdrawal volume, aquifer recharge from regional rainfall, irrigation and nonlinear deformation response due to the presence of surface soil. Further, it is difficult to constrain the surface subsidence due to very limited and relatively short duration of available cGPS measurements in the Delhi surrounding region (Fig.S9). To capture first-order effects and gain insights into the physical mechanisms using the limited data available, we consider a linear elastic homogeneous isotropic material model with a constant groundwater extraction rate. Yet, by honoring the two-way coupling between fluid flow and mechanical deformation processes, the model is sophisticated enough to investigate the relative significance of aquifer depletion-induced poroelastic effects, i.e. aquifer contraction and basement unloading, and its role in changing the stability of basement faults and associated seismicity around the Aravalli Delhi fold belt. This novel approach has been missing from some of the earlier attempts at modeling groundwater depletion-induced seismicity, which assumed undrained deformation of the basement and decoupling of mechanical deformation from fluid flow^1^.

**Figure 4:**
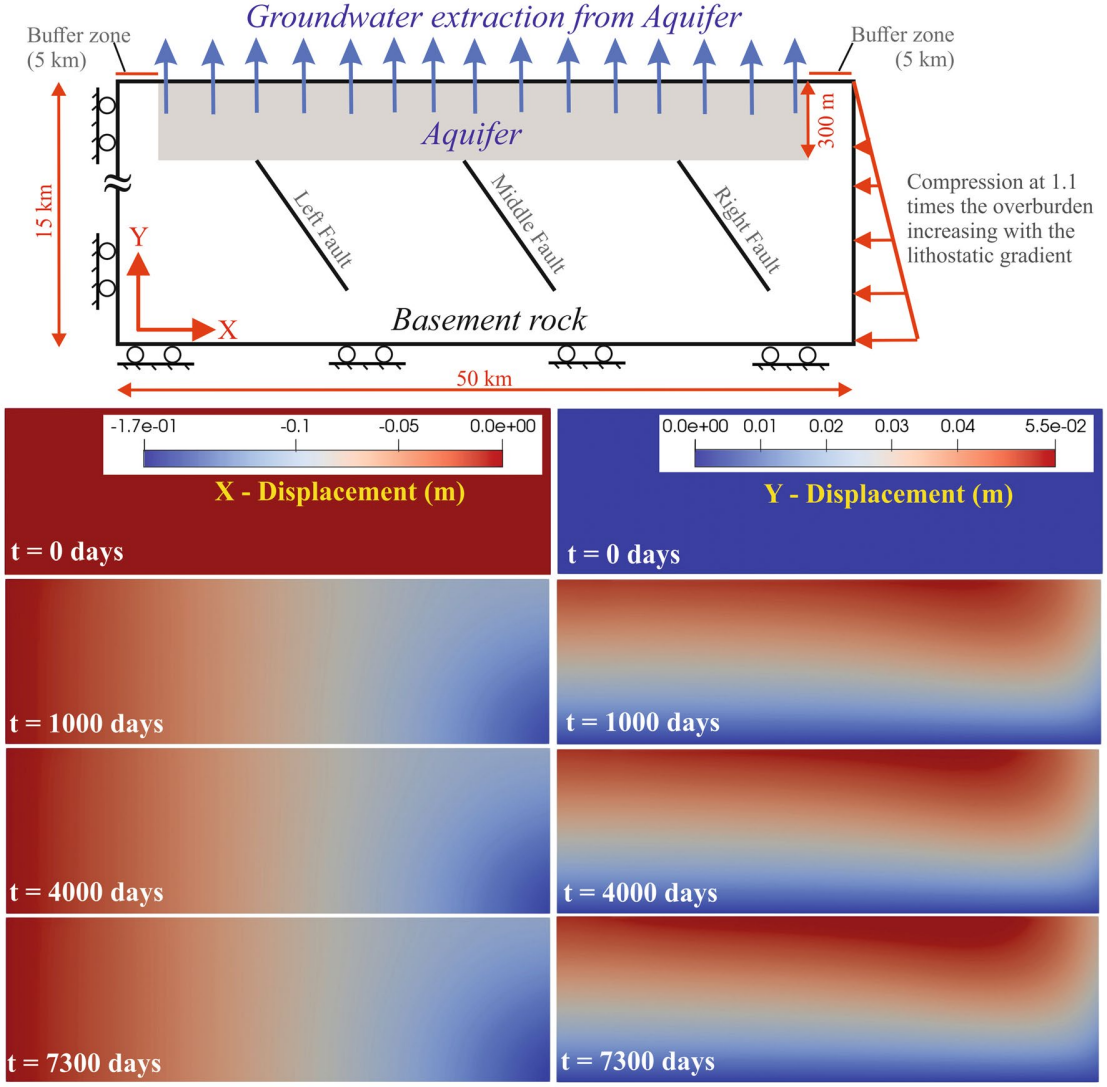
Geomechanical model of groundwater extraction-induced seismicity. The model geometry, dimensions and the boundary conditions are shown in the topmost panel. The unconfined aquifer is located within the top 300 m interval of the domain. Compression at 1.1 times the overburden weight is applied on the right boundary, the top boundary is traction‐free, and normal displacements on the other two boundaries are fixed to zero. A no‐flow boundary condition is imposed on all except the top boundary, which is a drained boundary with its pressure set at the atmospheric value. The X-displacement and Y-displacement in the entire domain due to groundwater extraction from the shallow aquifer are shown at four times steps in in the lower eight panes (see “Material and method”). This figure was generated using ParaView application (version 5.7.0 URL: https://www.paraview.org/).

We initialize the coupled model under a reverse faulting stress field (horizontal-to-vertical principal stress ratio of 1.1) and hydrostatic pore pressures to achieve the initial equilibrium in the system (described in “Methods”). The modeling geometry and boundary conditions are presented in Fig. 4 (top panel). We simulated groundwater extraction over two decades to obtain pressure and stress propagation profiles within the basement rock, and shear and normal tractions on a representative fault system that is assumed to be critically stressed under the imposed reverse faulting regime. Finally, we computed the combined effect of pressure depletion from the aquifer and basement unloading on the fault stability by evaluating the change in the Coulomb Failure Stress (CFS), which is defined as:

$$\Delta CFS=\left(\Delta \tau -\mu \Delta {\sigma }_{n}^{{\prime}}\right)=\Delta \tau -\mu (\Delta {\sigma }_{n}-\Delta p)$$

where $\Delta \tau$ is the change in the fault shear traction, $\Delta {\sigma }_{n}$ is the change in total normal traction (positive in compression),$\Delta {\sigma }_{n}^{^{\prime}}$ is the change in the effective normal traction,$\Delta p$ is the pore pressure change and $\mu$ is the coefficient of fault friction (assumed to be 0.6).

**Figure 5:**
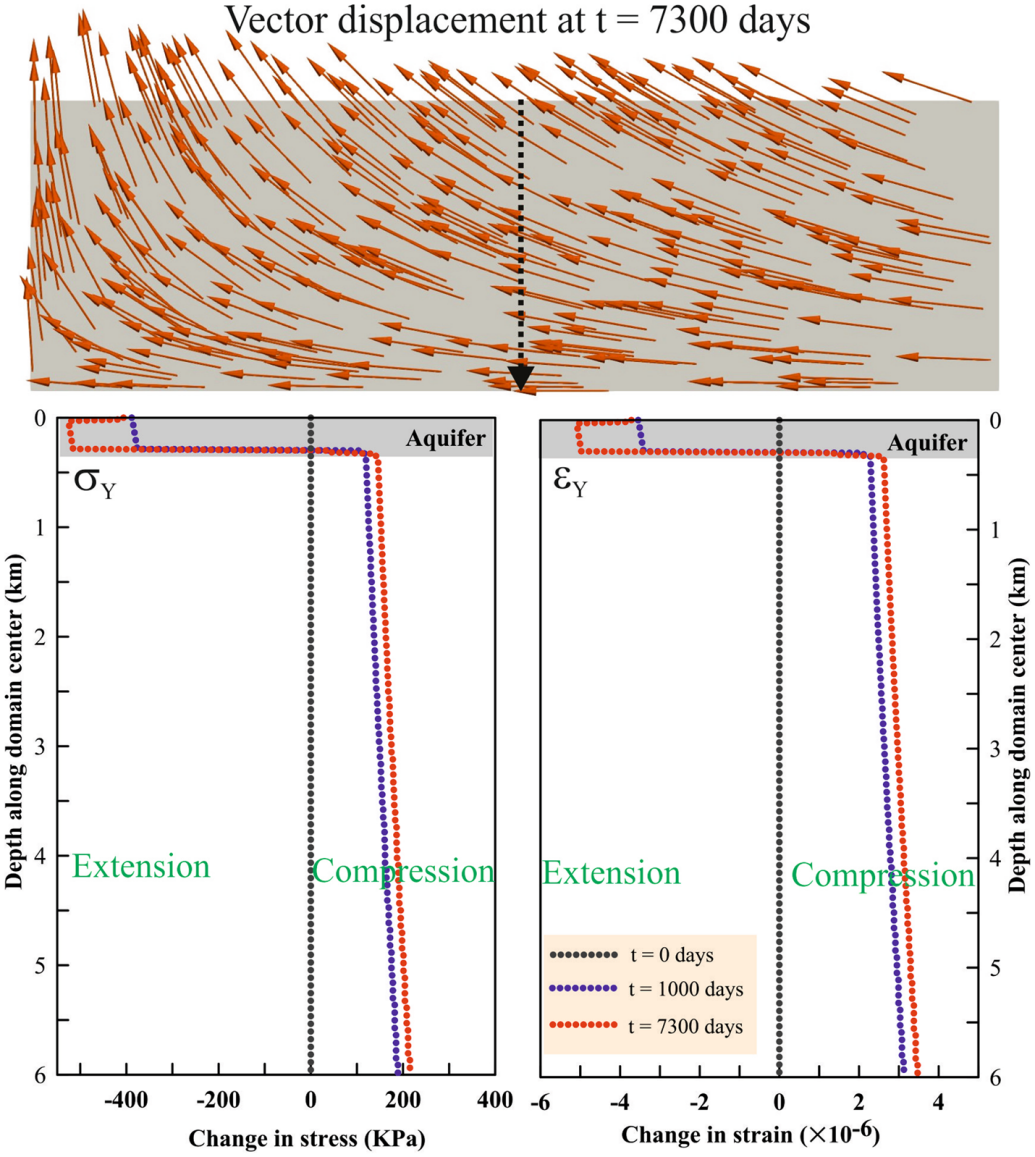
Aquifer contraction and basement rock expansion. Topmost panel shows the simulated displacement vector field after 20 years (i.e., 7300 days) of groundwater extraction from the aquifer. Its effect on aquifer contraction and basement rock expansion is shown by the corresponding stress and strain changes, which are plotted with depth along the domain center at different time steps in the lower two panes. This figure was generated using Grapher graphical application (version 8.7.844 URL: https://www.goldensoftware.com/products/grapher) and ParaView application (version 5.7.0 URL: https://www.paraview.org/).

**Figure 6:**
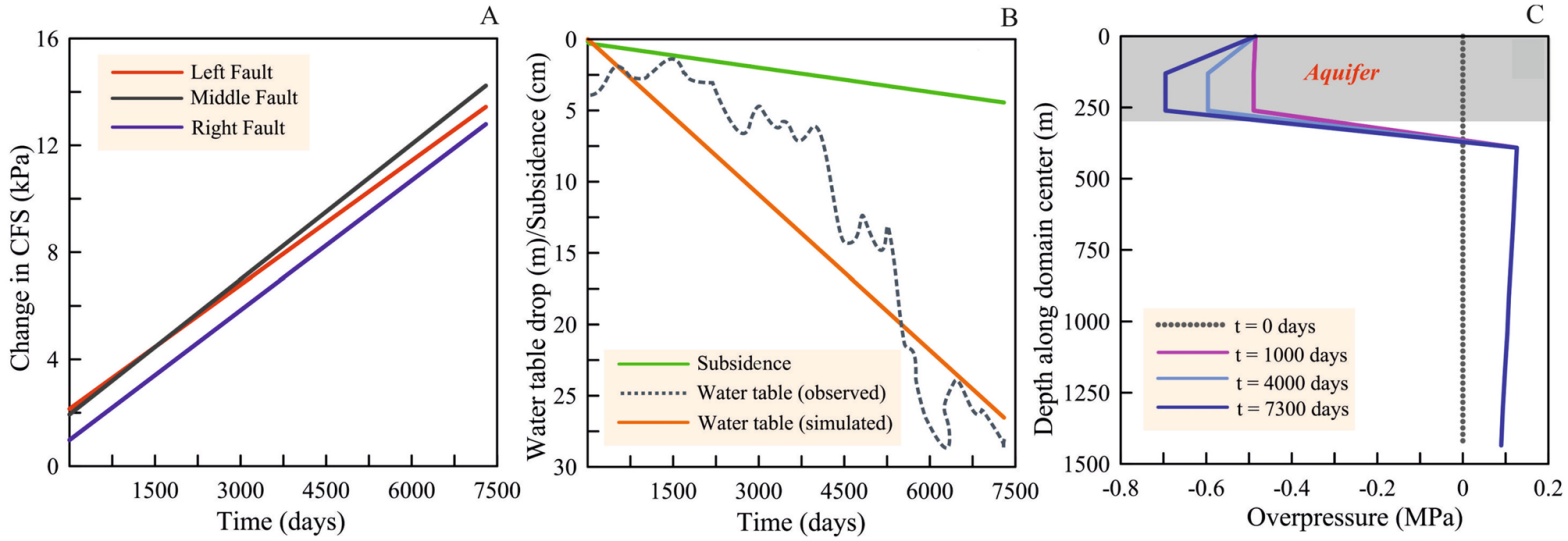
Fault stability, water table drop, ground subsidence and overpressure. **(A)** Change in fault stability due to water table drop is shown in terms of the change in Coulomb failure stress (CFS, in kPa) on the three faults at a depth of 10 km. Positive values correspond to destabilization and negative values correspond to stabilization. All faults experience destabilization under the reverse faulting mechanism. **(B)** Observed and simulated water table drop are consistent with each other. Ground subsidence increases at a rate of ~ 0.2 cm/year due to groundwater extraction. **(C)** Overpressure with depth along the model center at different times. Overpressure is shown over the 0–1.5 km depth interval which includes the 15 layers of the aquifer within 0–300 m interval (see also supplementary Figures). This figure was generated using Grapher graphical application (version 8.7.844 URL: https://www.goldensoftware.com/products/grapher).

From this coupled poromechanical simulation and associated fault stability analysis, we capture three important effects of long-term groundwater withdrawal in the Delhi surrounding region as follows (Figs. 4, 5,6): (a) First, the depletion of pore pressure leads to an increase in the effective compression and poroelastic contraction of the aquifer, which results into subsidence. Under the assumptions of small horizontal strains and uniaxial consolidation, subsidence can be approximated analytically as ${L}_{a}{\Delta p}_{a}/\left({K}_{dr}+4G/3\right)$, where ${L}_{a}$ is the aquifer thickness, ${\Delta p}_{a}$ is the aquifer pressure drop, ${K}_{dr}$ is the aquifer drained modulus and $G$ is the aquifer shear modulus. The depleted region increases laterally and vertically with time due to the effect of diffusion-induced pressure drop away from the wells, which is observed as the water table drops with time (Fig. 6B, Fig.S8). Hydraulic conductivity and porosity of the aquifer control the time scale and areal extent of ${\Delta p}_{a}$, and therefore, the poroelastic contraction effect. (b) The second effect is the unloading of the basemenst due to removal of the weight of water from aquifer leading to an elastic expansion of the basement. Under the elastic approximation, the change in the total stress due to groundwater extraction corresponds to the weight of water mass removed, which can be expressed by $\phi {\rho }_{w}A\Delta {z}_{wt}$, where $\phi$ is the aquifer porosity which changes during aquifer compaction, ${\rho }_{w}$ is the density of water (neglecting the density of air relative to that of water), $A$ is the aquifer area of unloading, and $\Delta {z}_{wt}$ is the drop in water table. Given the low conductivity of the basement, the undrained bulk modulus and the bulk density control the time scale of the elastic expansion effect. (c) The third effect is the pressure drop in the basement due to propagation of the pressure diffusion front into basement and elastic expansion of the basement. A low value of basement conductivity indicates weak diffusion and an undrained pressure drop that can be approximated with $B\Delta {\sigma }_{v}$, where $B$ is Skempton’s coefficient and $\Delta {\sigma }_{v}$ is the change in the total volumetric stress.

The relative strengths of the above three effects change with time as groundwater pumping continues, and these effects cannot be captured accurately in uncoupled models that solve the mechanical equilibrium problem with a pore pressure field that was pre-computed using a diffusion model. For example, the effect of aquifer pressure diffusion may penetrate into basement and drive the contraction–expansion boundary deeper with time as shown in Fig. 4, Fig.S7. This is a dynamic effect that can only be captured through a coupled poromechanical model and is missed in uncoupled models or one-way coupled models that assume undrained deformation (one-way coupling implies no coupling from mechanics to fluid flow).

Both aquifer contraction and basement expansion effects act together to modulate the effective stress regime on the basement faults (as presented in Fig. 5, Fig.S5, Fig.S6). Figure 6A represents relative fault stability (i.e., by $\Delta CFS$ kPa) due to water table drop over the period of two decades (Fig. 6B). It has been observed that basement faults experience destabilization under the reverse faulting stress regime, however the change in effective normal traction varies based on the fault location with respect to the source of unloading, i.e. the aquifer. Therefore, the middle fault experiences a slightly higher rate of destabilization than the other two faults (Fig. 6A). The cumulative change in $\Delta CFS$ on the basement faults of ~ 12–15 kPa, is much lower in magnitude compared to the stress drop estimated from the seismic energy released during the earthquakes, but well above the critical threshold for seismic triggering^35–37^. Observed and simulated water table drop over the period of last two decades are consistent with each other (Fig. 6B). Moreover, simulated surface subsidence rate (0.2 cm/yr) appears to be consistent with the sparsely located geodetic subsidence rates (~ 0.3 to 0.1 cm/yr) of the Delhi surrounding region (Fig.S9), although we acknowledge that the spatiotemporal discrepancy in subsidence rates may be attributed to the assumption of a homogeneous elastic rheology in our model (Fig. 6B). Agreement between the predicted and expected depth profiles of pressure change due to groundwater extraction further increases confidence in model’s calibration and prediction potential (Fig. 6C, Fig.S7). We further acknowledge that our coupled poromechanical model is limited and is mostly qualitative in nature because it does not consider any uncertainties in the poroelastic properties, which could change the spatial and temporal distribution of the results. In fact, we used constant values of poroelastic properties and did not consider stress-dependent properties due to the lack of availability of data on stress-dependency of moduli in the Delhi and surrounding region. Nevertheless, it is a common practice in modeling studies to prefer simplicity over complexity in the simulation^38^.

## Discussion

**Figure 7:**
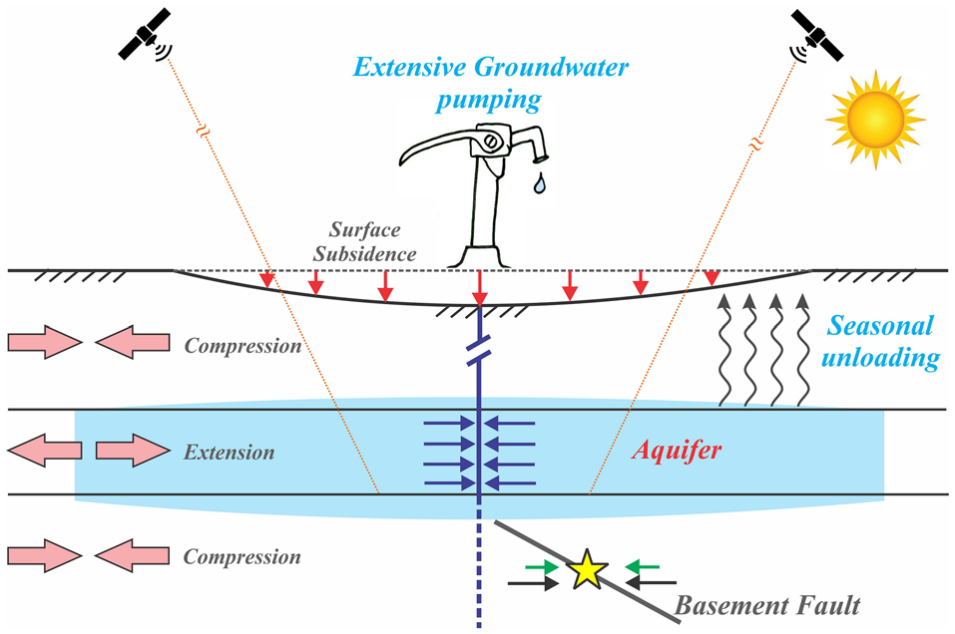
Schematic representation of crustal unloading, ground subsidence, and faulting associated with groundwater extraction from unconfined aquifer and seasonal unloading. The light blue color region indicates depressurized zone within the aquifer due to extensive groundwater extraction from unconfined aquifer. Compression of basement rocks near extensive pumping favors reverse faulting. Crustal unloading due to groundwater extraction and seasonal unloading cause basement expansion which can apply horizontal compression (green arrows) on bounding faults that are far from the aquifer. This compression adds to the secular long-term contraction (black arrows). Yellow star represents hypothetical hypocenter of seismicity at the basement faults. This figure was generated using Corel Draw graphical application (version 18 URL: https://www.coreldraw.com/en).

Based on the data analysis and model results, we propose that the seismicity in the Aravalli Delhi fold belt has possibly been influenced by the non-tectonic anthropogenic groundwater pumping-induced fault destabilization process and seasonally controlled hydrological unloading from the alluvium aquifer, although the mechanism of faulting itself is a tectonically driven phenomenon (Fig. 7). We acknowledge that the Aravalli Delhi fold belt associated seismicity occurred on the basements faults, beneath the aquifer, predominantly in response to the regional tectonic loading process. However, the exact rate of tectonic stress accumulation is unknown due to a lack of geodetic constrains in this plate interior domain. We argue that, besides the ongoing tectonic loading process on the basement faults, a significant component of horizontal compression was added over past several decades to the secular interseismic compression at seismogenic depth, due to extensive extraction of groundwater and the seasonal unloading process (Fig. 7).

### Higher seismicity in the Delhi region

It has been argued that the seismicity in the Delhi region is generally higher in comparison to that of the entire Aravalli Delhi fold belt^17^. They ascribed it to the strong seismic network in the Delhi region. However, here we propose that it could also be due to the higher rate of water extraction from the alluvial plains of the Delhi and adjoining northern India regions, in comparison to the region south of it, in the Aravalli Delhi fold belt where the groundwater extraction rate is lower (Fig. 1B). Thus, higher water extraction rate in the north causes higher seismicity in Delhi region. Moreover, the seismicity rate in Delhi region changes with the rate of water extraction.

### Any triggering thresholds for human-induced earthquake?

This present finding certainly raises an important question about the physics behind the triggering phenomenon and its critical triggering thresholds for Human-induced earthquake. Critical stress perturbations reported in the literature are as follows: 0.1 − 10 kPa for seismic waves^35,36^, 0.15 − 0.3 kPa for tides^37^, about 0.05 − 0.15 kPa/year for anthropogenic groundwater unloading such as in the case of 2015 Gorkha earthquake in Nepal^6^ and ~ 0.16 kPa/year in the 2017 Iran–Iraq border earthquake^7^. Climatic induced stress perturbations are negligible fractions of the stress drop during large earthquakes on plate boundary (or plate interior) regions, still it is sufficient to resonate the critically stressed fault system^39–41^. Further, it has also been argued that no lower threshold exists for earthquake triggering in central California, as the faulting geometry are random and earthquakes would have equal probabilities of being delayed or triggered^42^. In view of the above estimates, we argue that our estimated Coulomb failure stress change induced by extensive pumping of groundwater from the Delhi surrounding aquifer is close to the triggering threshold for seismicity. Although, we acknowledge that it is difficult to comment on the relative contributions of tectonically driven, seasonal hydrological-driven, and groundwater pumping-induced stress perturbations.

### Complementing other human-induced earthquakes

So far, more than 700 earthquake examples have been reported in the Human-induced earthquake Database, *HiQuake*^4,5^. However, many documented reports on anthropogenic groundwater pumping-induced stress perturbation and basement fault stability do not perform a thorough investigation of causality between the two processes because such an investigation REQUIRES a multi-scale, multiphysics simulation framework that is still an area of active research in computational geomechanics. Using an analytical modeling approach, groundwater extraction in San Joaquin Valley, California has been linked to the seismicity of the region^3^. An identical approach has been adopted for explaining the catastrophic April 2015 M7.8 Nepal Gorkha earthquake due to anthropogenic pumping of groundwater in the Indo-Ganga plains^6^. The 2011 M5.1 Lorca Spain earthquake due to groundwater extraction in the Alto-Guadalentin basin^1^ and the 2017 November M7.3 Iran–Iraq border earthquake due to groundwater pumping and crustal unloading in the Euphrates and Tigris River plains and western Iran^7^ are other representative examples. Further, fluid-injection-induced earthquake sequence from 2013 to 2015, recorded by the Colombian Geological Survey, in the municipality of Puerto Gaitán (Colombia) has been linked with the structural high, where the crystalline basement is superficial and Bouger gravity anomaly is high^43^.

An increasing number of natural observations associated with induced seismicity suggests that managing fluid injection rates may be a promising tool to mitigate the occurrence of induced earthquakes. In fact, it has been reported that low-rate wells, for instance, are much less likely to be associated with earthquakes than high-rate wells, and that the critical rate above which earthquakes are induced, is likely dependent on reservoir properties^44,45^. It has also been highlighted that the temporal variation in injection rates is generally correlated with the frequency of earthquakes^46–49^, and that abrupt increases in injection rates tend to shortly precede the occurrence of earthquakes^50–53^. Indeed, this is also the case here where the earthquakes of Delhi and surrounding region (i.e., induced seismicity zone) appear to be associated with the region of maximum depletion rate ( 1.6 cm/year, Fig. 1).

In order to assess objective seismic hazard in an induced seismicity zone e.g. induced seismicity region in oil or gas^14,54,55^ fields or induced seismicity in geothermal systems^56^, the maximum magnitude of observed induced seismicity is generally proportional to the fluid volume injected or extracted. In comparison to wastewater disposal wells, geothermal operations involve less fluid volume resulting in smaller size earthquakes^56^. The Delhi region has a longer history of earthquakes with reliable magnitude estimates, however an estimate of spatio-temporal rate of groundwater extraction volume is unknown in the region, which is generally known in cases of induced seismicity in hydrocarbon or geothermal systems^14,56^. Hence, unlike the cases of hydrocarbon or geothermal systems, here it is difficult to correlate the maximum magnitude with the volume of water extracted.

While complementing these earlier studies, our study presents a novel approach to explain the Human-induced seismicity around the Aravalli Delhi fold belt as well as the mechanical stability of the basement faults hosting such events. The novelty of the approach lies in the integration of multiphysics modeling with geodetic and hydrologic data analysis for an objective assessment of the seismic hazard in an actively developing, environmentally sensitive region in the world. Delhi, which is the capital city of India and heavily populated, can represent other mountain valley regions in the world that have experienced overexploitation of their aquifers, due to extensive irrigation, urban development, low/fluctuating rainfall, and suffered from ground subsidence and seismicity. The two-way coupling between fluid flow and mechanical deformation processes in our approach also holds promise for uncovering hidden basement faults and effective forecasting of Human-induced earthquakes.

## Methods

### Groundwater storage change

We quantified the annual groundwater storage anomalies by using the water balance method. Over 15 years of Gravity Recovery and Earth Climate Experiment (GRACE) measurements of total terrestrial water storage changes^7,57–60^ and global land data assimilation system (GLADS)^61^ water content data were used. In this computation, we have used recent GRACE data (1° × 1°) provided by the National Aeronautics and Space Administration’s (NASA) Jet Propulsion Laboratory (JPL) (https://grace.jpl.nasa.gov/). We have considered GRACE RL05 data, which is based on spherical harmonics solutions of JPL^62^. Further, we have used monthly of NASA’s GLDAS four vertical levels of soil moisture data of 1° × 1° spatial resolution^58,61^ archive at https://disc.gsfc.nasa.gov/datasets?keywords=GLDAS, in order to obtain the primary land surface flux and storage component for the same time period of GRACE observation.

The groundwater storage changes of the total terrestrial water storage can be estimate from the GRACE and this has expressed as:

1 $$\Delta GW =\Delta S -\Delta SWE -\Delta SW -\Delta SM$$

where $\Delta$ GW is the groundwater storage changes, $\Delta$ S, $\Delta$ SWE, $\Delta$ SW, $\Delta$ SM represent total terrestrial water storage, snow water equivalent, surface water storage, soil moisture changes respectively. The anomalies of snow water equivalent, surface water storage and soil moisture changes are extracted from the NASA Noah land surface model. Monthly time-series of $\Delta$ GW are used to determine the statistical significance of the trend by employing the non-parametric Mann–Kendall test^63,64^. Presented results are significance at the 95% confidence level (p < 0.05). Further, based on the respective errors of the specific components, the trend error in groundwater change is estimated using expression below:

2 $${\sigma }_{GW}=\sqrt{{({\sigma }_{S})}^{2}+{({\sigma }_{SWE})}^{2}+{({\sigma }_{SW})}^{2}+{({\sigma }_{SM})}^{2}}$$

where, $\sigma$ represents one-sigma trend errors in the corresponding components.

## Coupled poro-mechanical model and fault stability

### (a) Governing equations & mathematical formulation

The governing equations in our coupled simulation framework are the fluid mass conservation equations for the air and water phases and the solid linear momentum balance equations in 2-D^65–68^. Under the quasi-static equilibrium assumption, the linear momentum balance is given as

3 $$\nabla \cdot{\varvec{\sigma}}+{\rho }_{b}{\varvec{g}}=0$$

where σ is the Cauchy total stress tensor, ***g*** is the gravity vector, ${\rho }_{b}=\phi \left({\rho }_{a}{(1-S}_{w})+{\rho }_{w}{S}_{w}\right)+\left(1-\phi \right){\rho }_{s}$ is the bulk density, ${\rho }_{a}$ is the air density, ${\rho }_{w}$ is the water density, ${\rho }_{s}$ is the solid grain density, ${S}_{w}$ is the water saturation, and $\phi$ is the porosity that evolves with time as per the solid mass conservation equation. The mass conservation for fluid phase $\beta$ (water or air) can be written as:

4 $$\frac{d{m}_{\beta }}{dt}+\nabla \cdot {{\varvec{w}}}_{\beta }={\rho }_{\beta }{f}_{\beta },$$

where the accumulation term $d{m}_{\beta }/dt$ describes the time variation of fluid mass relative to the motion of the solid skeleton, ${{\varvec{w}}}_{\beta }$ is the mass-flux of fluid phase $\beta$ relative to the solid skeleton, and ${f}_{\beta }$ is the volumetric source term for phase $\beta$ due to pumping wells in the aquifer. Mass (per unit bulk volume in the reference configuration) of phase $\beta$ is ${m}_{\beta }={\rho }_{\beta }{S}_{\beta }\phi \left({1+\epsilon }_{v}\right)$, where the volumetric strain ${\epsilon }_{v}$ is related to the change in effective volumetric stress ${\sigma }_{v}^{^{\prime}}$ as ${\epsilon }_{v}={\sigma }_{v}^{^{\prime}}/{K}_{dr}$ assuming a linear elastic material (prior to failure) with the drained bulk modulus ${K}_{dr}$ given in terms of the drained Young’s modulus *E* and Poisson’s ratio *ν.* The fluid phase saturations sum to $1.$ Flux ${{\varvec{w}}}_{\beta }$ is related to the phase pressure ${p}_{\beta }$, density ${\rho }_{\beta }$, viscosity ${\mu }_{\beta }$, and relative permeability ${k}_{r\beta }$ through the multiphase extension of Darcy’s relation. Relative permeabilities are expressed as functions of ${S}_{w}$ using the Brooks-Corey model^38,67^. The effective stress tensor ${{\varvec{\sigma}}}^{^{\prime}}$ is given in terms of the total stress tensor and pressure as ${{\varvec{\sigma}}}^{\boldsymbol{^{\prime}}}={\varvec{\sigma}}+bp1$ (normal stresses assumed positive in tension) where $1=[1, 1, 0]$ is the 2-D identity vector in the engineering notation and $p={p}_{w}+\left(1-{S}_{w}\right){P}_{c}$ is the saturation-weighted equivalent pore pressure, where ${P}_{c}={p}_{a}-{p}_{w}$ is the capillary pressure modeled here as an analytical function of water saturation ${S}_{w}$ using van Genuchten’s model^67^**.** $b$ is the Biot Coefficient, which accounts for the compressibility of solid grains relative to that of the porous skeleton. Assuming homogeneous isotropic linear elastic behavior of the medium, ${{\varvec{\sigma}}}^{{{\prime}}}={\varvec{D}}{\varvec{\epsilon}}$, where ${\varvec{D}}$ is the drained elasticity tensor given in terms of the drained Young’s modulus *E* and Poisson’s ratio $\nu$, both related to *K*_*dr*_. The linearized strain tensor ${\varvec{\epsilon}}$ is defined as the symmetric gradient of the displacement vector ${\varvec{u}}$ as ${\varvec{\epsilon}}={\nabla }^{\mathrm{sym}}{\varvec{u}}$. The volumetric strain is given as ${\epsilon }_{v}=\nabla \cdot {\varvec{u}}$.

The constitutive equation of poroelasticity for fluid mass increment can be expressed as for phase $\beta$

5 $$\frac{d{m}_{\beta }}{{\rho }_{\beta }}=\left(\frac{b}{{K}_{dr}}d{\sigma }_{v}+\frac{{b}^{2}}{{K}_{dr}}dp\right){S}_{\beta }+\left({{N}_{ww}dp}_{w}+{{N}_{wa}dp}_{a}\right)$$

where ${N}_{ww}$ and ${N}_{wa}$ are the water-water and water–air inverse Biot moduli that are functions of the rock and fluid compressibilities and the fluid saturations. The inverse Biot moduli are components of the inverse Biot modulus tensor ***N* = *M***^*1*^ where ***M*** is the multiphase Biot modulus tensor. For single-phase flow with a fluid compressibility ${c}_{f}$, the inverse Biot modulus is expressed as $\frac{1}{M}= \phi {c}_{f}+\frac{\left(\alpha -\phi \right)\left(1-\alpha \right)}{{K}_{dr}}$. Equation (5) relates the increment in fluid mass ($d{m}_{\beta }$) to the change in total volumetric stress $d{\sigma }_{v}$ and the change in the fluid pressures $dp$. Substituting this equation in Eq. (4) produces two pressure equations for the two fluid phases (water and air). The coupled flow-geomechanics pressure equation is different from the uncoupled (flow-only) pressure equation in two important ways: (1) a $\partial {\sigma }_{v}/\partial t$ term appears in the coupled equation that accounts for two effects of extraction on pressure: pore contraction in the depleted region near pumping wells and rock expansion in non-depleted region away from the wells, and (2) the rock compressibility term that multiplies $\partial {p}_{\beta }/\partial t$ in the flow-only equation is now replaced by a term that combines $b,{K}_{dr},$ and ${\varvec{N}}$, which truly govern the time-dependent compressibility of the rock-fluid system. Here, we assume constant values of these poroelastic properties, and we neglect stress-dependency of poroelastic moduli. This is due to a lack of data availability and the need to keep the model simpler. The mathematical formulation is two-way coupled: fluid flow affects mechanical equilibrium through pressure-induced changes in the effective stress and fluid density-induced changes in the gravitational body force, and mechanical deformation affects fluid flow through the rate of volumetric stress, which induces changes in the pore volume and pressure.

### (b) Solution approach

We discretize the fluid mass balance equation using an implicit finite volume method with piecewise constant element-centered pore pressures and water saturations. The Backward Euler method is used for time integration. This method is second-order accurate in space and first-order accurate in time. We discretize the mechanical equilibrium equation using the Galerkin finite element method with nodal displacement vectors on bilinear elements. This method is second-order accurate in space. We use an unconditionally stable sequential iterative solution scheme^66,67,69^, to solve the coupled system of discretized equations sequentially and iteratively till convergence at each time step (Fig. S4). In this scheme, flow and geomechanics sub-problems are solved using their respective Newton solver.

### (c) Modeling geometry and boundary conditions

The modeling geometry, boundary conditions and physical parameter used in this coupled poro-mechanical model are presented in the top panel in Fig. 4 and Table 1. The normal displacement is prescribed to be zero on the left and bottom boundaries of the domain. The top boundary at the ground surface is a traction-free boundary. The right boundary has a compression that is 1.1 times the overburden (multiple modeling studies have considered the ratio of maximum to minimum principal stresses in the range of 1–2 in a thrust-faulting stress regime, which is based on worldwide stress map^8,70^), which increases with depth following the lithostatic gradient. All boundaries except the top boundary are considered as no-flow boundaries (Fig. 4). We model the top boundary as a prescribed pressure boundary with the pressure value set to be equal to the atmospheric pressure. Water is withdrawn using 50 pumping wells placed approximately uniformly in the aquifer. Wells are open to the aquifer in the top four layers of the grid (*z* < 300 m). They are producing with prescribed flow rates during 1985–2005 time period. We include three representative faults in the basement rock beneath the aquifer, which correspond to the existing earthquake focal mechanism in the region. The faults are dipping ~ 60° and differ in their relative position with respect to the aquifer. We create a geomechanical domain that is large enough compared to the size of the aquifer to limit the numerical artifacts due to the mechanical boundary conditions (compression and fixed displacement) imposed on the two lateral boundaries. We place the vertical boundaries of the geomechanical domain at a distance of 5 km from the edges of the aquifer. Within this buffer zone of 5 km on each side, the hydraulic permeability is set to a relatively low value compared to the aquifer permeability. This limits the propagation of aquifer pressure perturbation to the lateral boundaries of the domain, which limits the numerical artifacts of the boundary conditions on the simulation results (pressure, displacements, and fault stresses). In particular, the buffer zone limits the magnitude of artificial tensile stresses created near the fixed displacement left boundary and the bending moment near the top right corner of the domain where the traction boundary conditions of the top and right boundaries meet.

**Table 1:** Parameters used in the coupled poro-mechanical model.

Poisson’s ratio	0.26
Shear modulus, *G*	27.21 GPa
Solid density, *ρ*_*s*_	2500 kg/m^3^
Water density, *ρ*_*w*_	1037 kg/m^3^
Aquifer permeability	500 mD
Basement permeability	0.001 mD
Initial aquifer porosity	0.1
Fault permeability	10^–6^ mD
Biot coefficient	0.8
Drained bulk modulus, *K*_*dr*_	48.5 GPa

### Physical parameters

See Table 1.

## Supplementary Information

Supplementary Figures.

## Data Availability

All the data and computational code used in these present analyses are available openly at public domain and presented in the text and supporting documents. Precipitation data used in this paper can be found at Global Precipitation Climatological Centre (GPCC, http://www.esrl.noaa.gov/psd/). GPS time series from the Delhi surrounding region can be archived from http://geodesy.unr.edu/NGLStationPages/gpsnetmap/GPSNetMap.html. We used our in-house coupled flow and geo-mechanical simulator that is based on *PyLith* (www.geodynamics.org/cig/software/pylith/) for poromechanical model and fault stability analysis. The earthquake data from NCS are available at https://seismo.gov.in/. All the other relevant data are available from the corresponding author upon reasonable request.
