# Supplementary material for: Groundwater extraction-induced seismicity around Delhi region, India
Source: Sci Rep. 2021 May 12;11:10097. doi: 10.1038/s41598-021-89527-3 (PMC8115671; doi:10.1038/s41598-021-89527-3)
Supplement: Supplementary file 1 — Supplementary Figures. [file 41598_2021_89527_MOESM1_ESM.docx]

**Supporting documents**

Groundwater extraction-induced seismicity around Delhi region, India

**Deepak K Tiwari^1^, Birendra Jha^2^, Bhaskar Kundu^1*^, Vineet K Gahalaut^3^, Naresh K. Vissa^1^**

**^1^**Department of Earth and Atmospheric Sciences, NIT Rourkela, Rourkela 769008, India

**^2^**Department of Chemical Engineering and Materials Science, University of Southern California, Los Angeles 90007-1211 CA, USA

**^3^**CSIR-National Geophysical Research Institute, Uppal Road, Hyderabad 500007, India

**^*^Corresponding author:** Bhaskar Kundu, Department of Earth and Atmospheric Sciences, NIT Rourkela, Rourkela-769008, India, ([rilbhaskar@gmail.com](mailto:rilbhaskar@gmail.com)).

**Supporting Figures:**

**
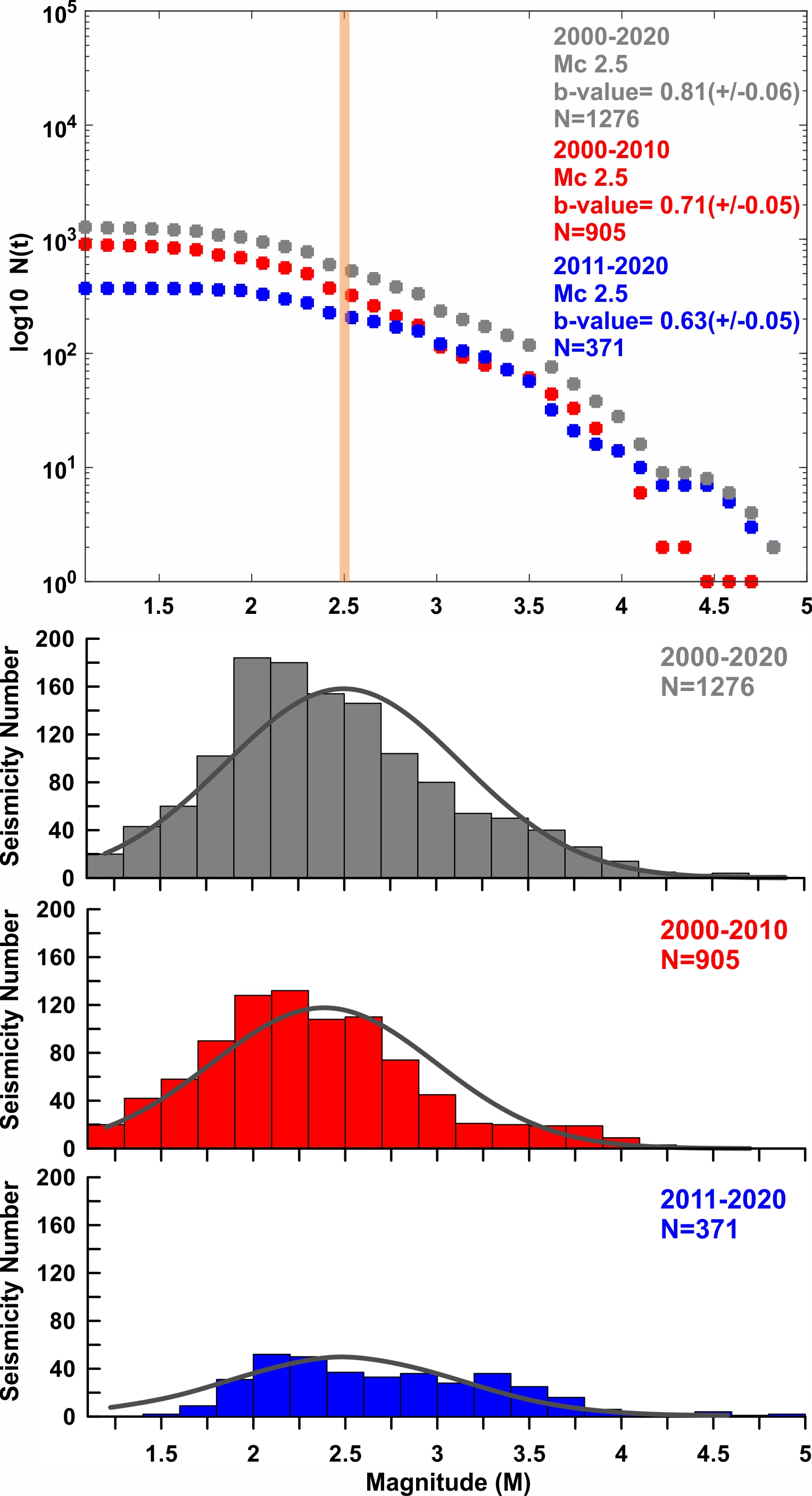
**

**Figure S1:** Magnitude completeness (Mc) of seismicity over Delhi region with different time periods. Note that the overall Mc is constant i.e., 2.5 over different time period. This figure was generated using Grapher graphical application (version 8.7.844 URL: <https://www.goldensoftware.com/products/grapher>).


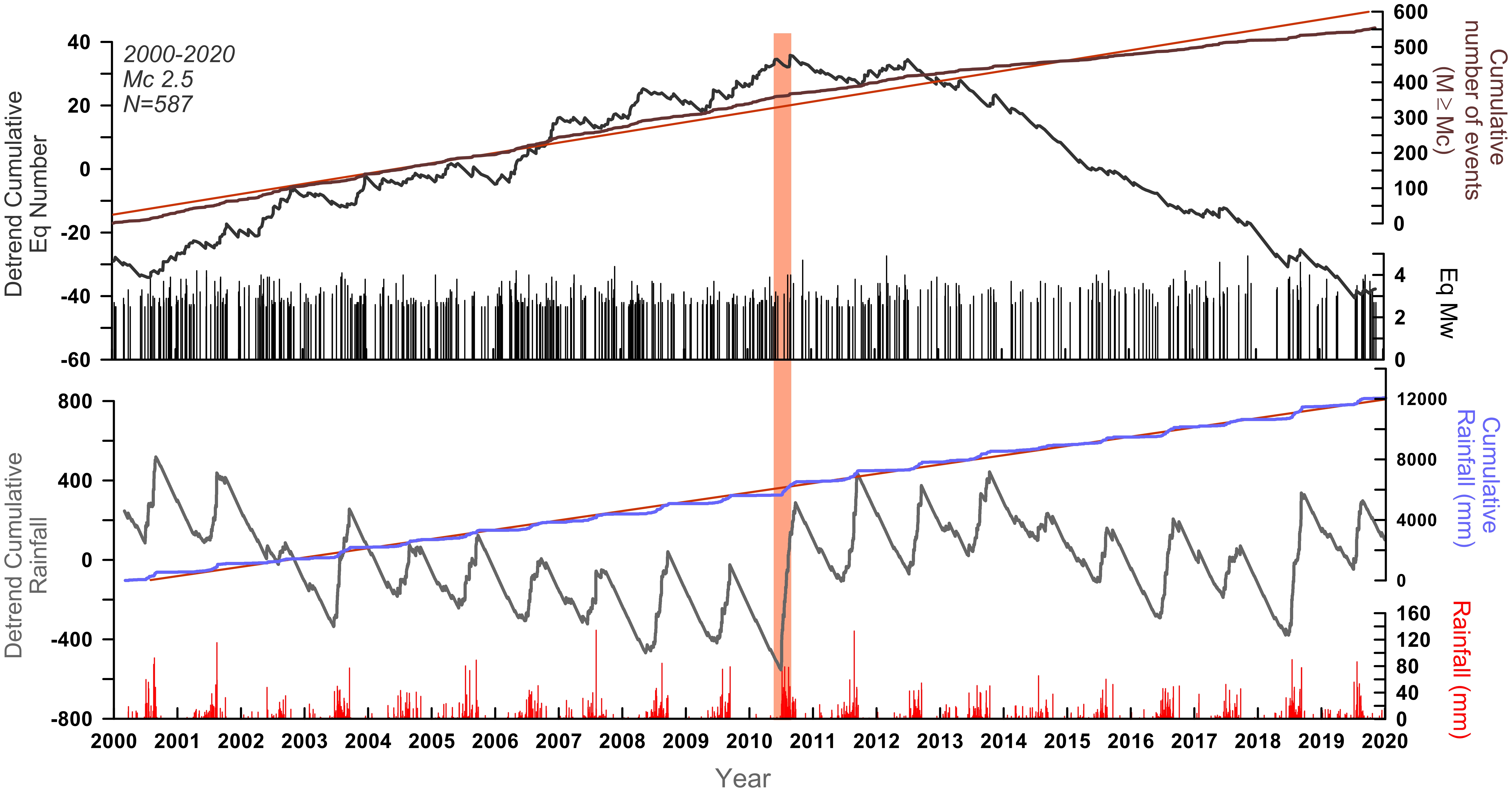


**Figure S2:** Rainfall and seismicity distribution (Mc 2.5) along with respective detrend residuals. The red line in each case denote the best fitting line in the cumulative trend. Note that after 2011 there is a decrease in seismicity frequency while the amount of rainfall shows an increasing trend. This figure was generated using Grapher graphical application (version 8.7.844 URL: <https://www.goldensoftware.com/products/grapher>).


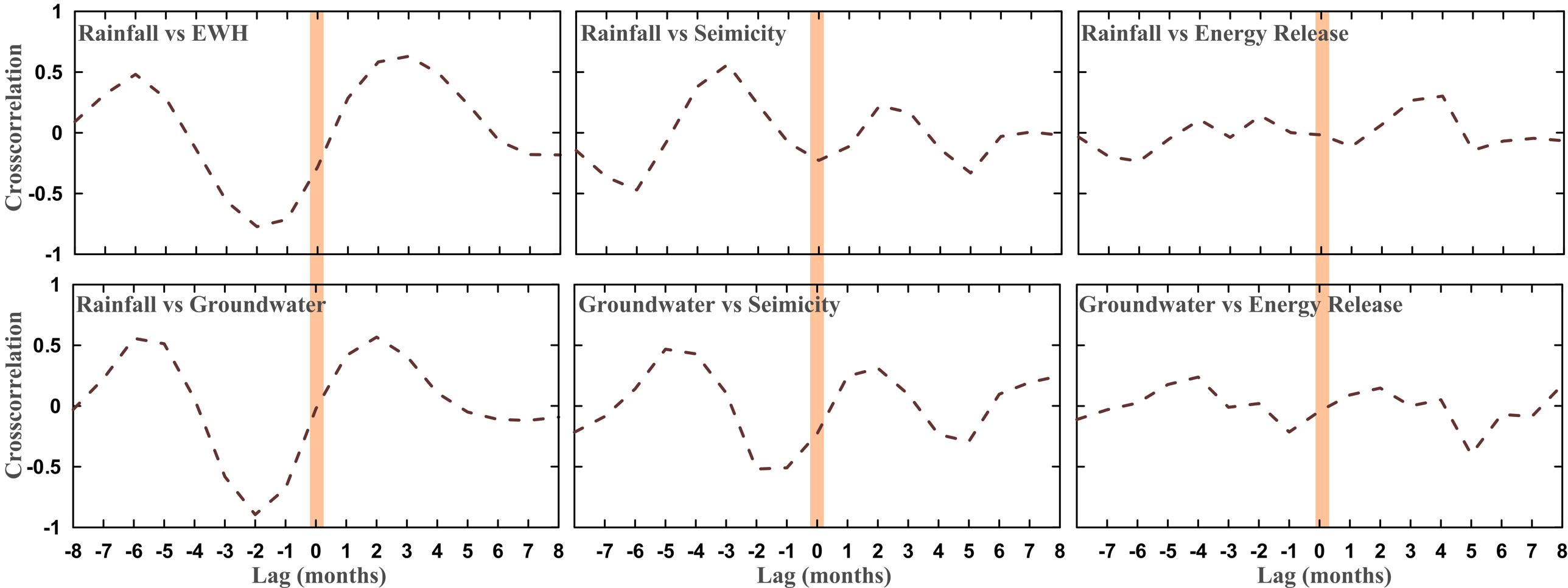


**Figure S3:** Cross-correlation among various physical parameters (between rainfall vs GRACE derived EWH, seismicity, energy release and groundwater) This figure was generated using Grapher graphical application (version 8.7.844 URL: <https://www.goldensoftware.com/products/grapher>).


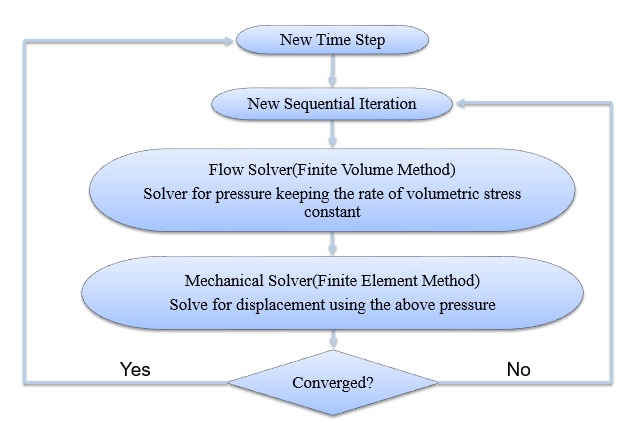


**Figure S4:** The sequential iterative solution scheme used in our coupled flow geomechanical simulator. This figure was generated using Corel Draw graphical application (version 18 URL: <https://www.coreldraw.com/en>).

**
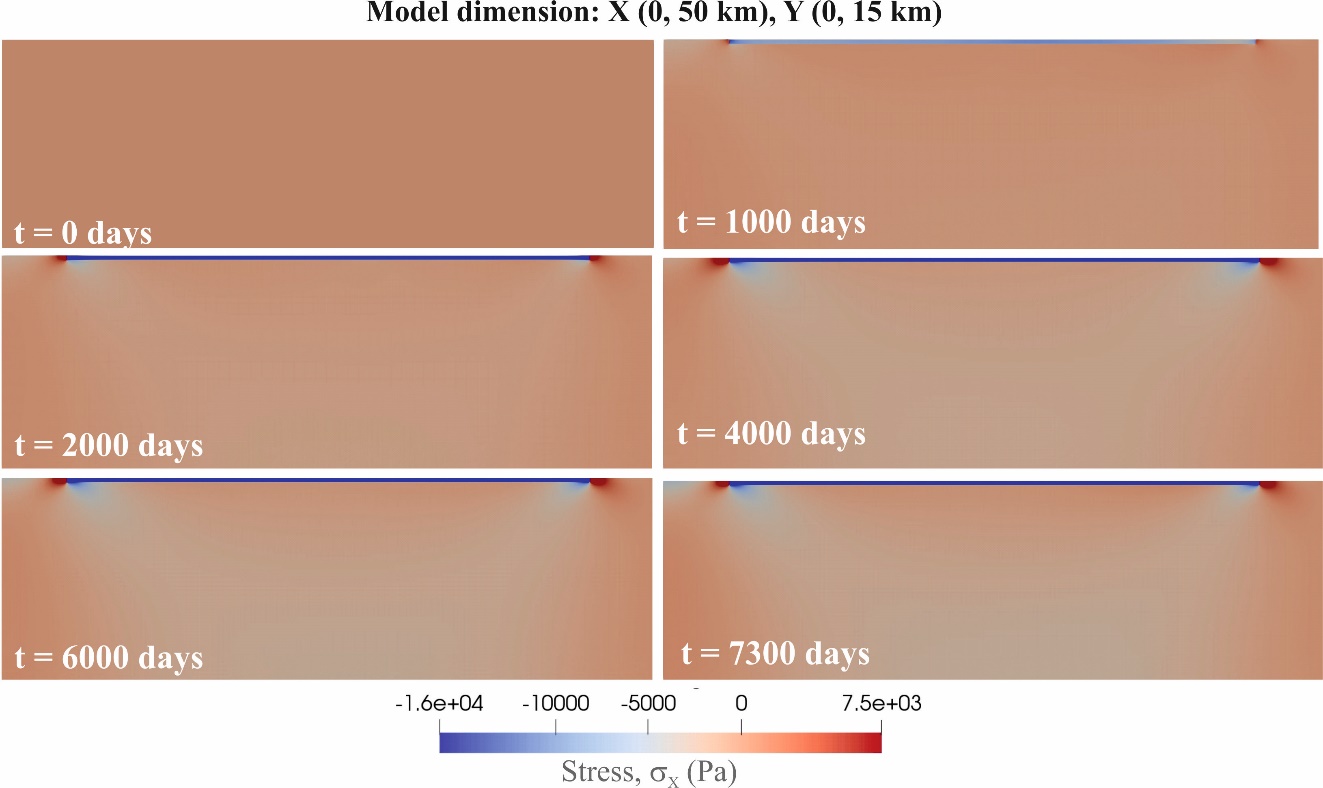
**

**
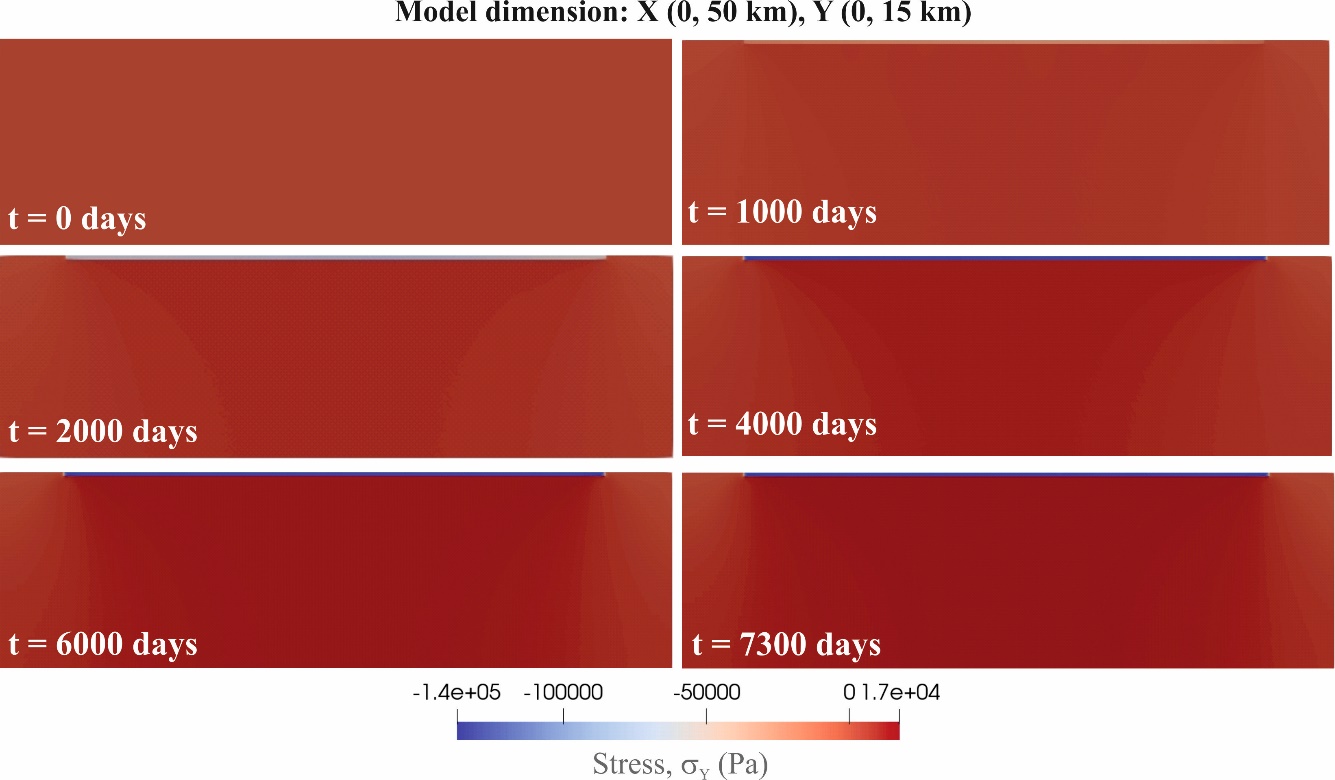
**

**Figure S5:** Groundwater extraction-induced changes in the stress components σ_x_ and σ_Y_ at six different time steps. This figure was generated using ParaView application (version 5.7.0 URL: <https://www.paraview.org/>).

**
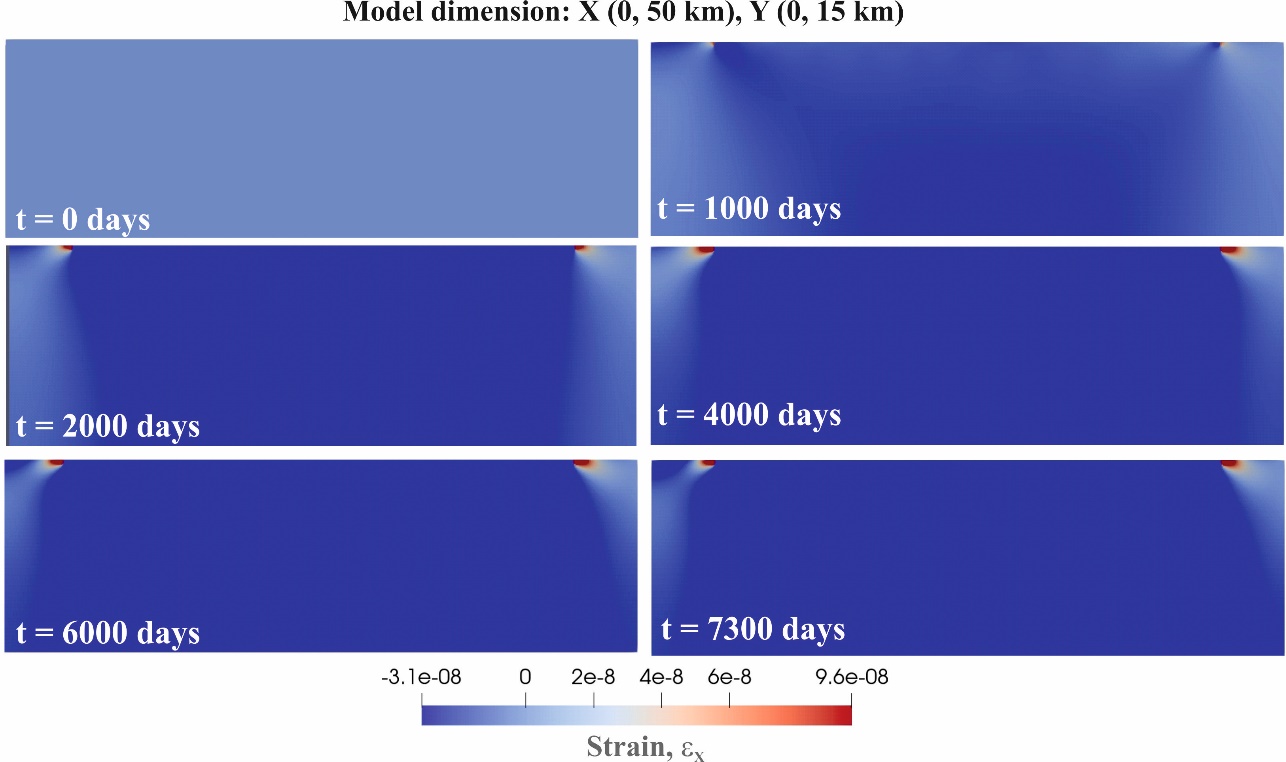
**


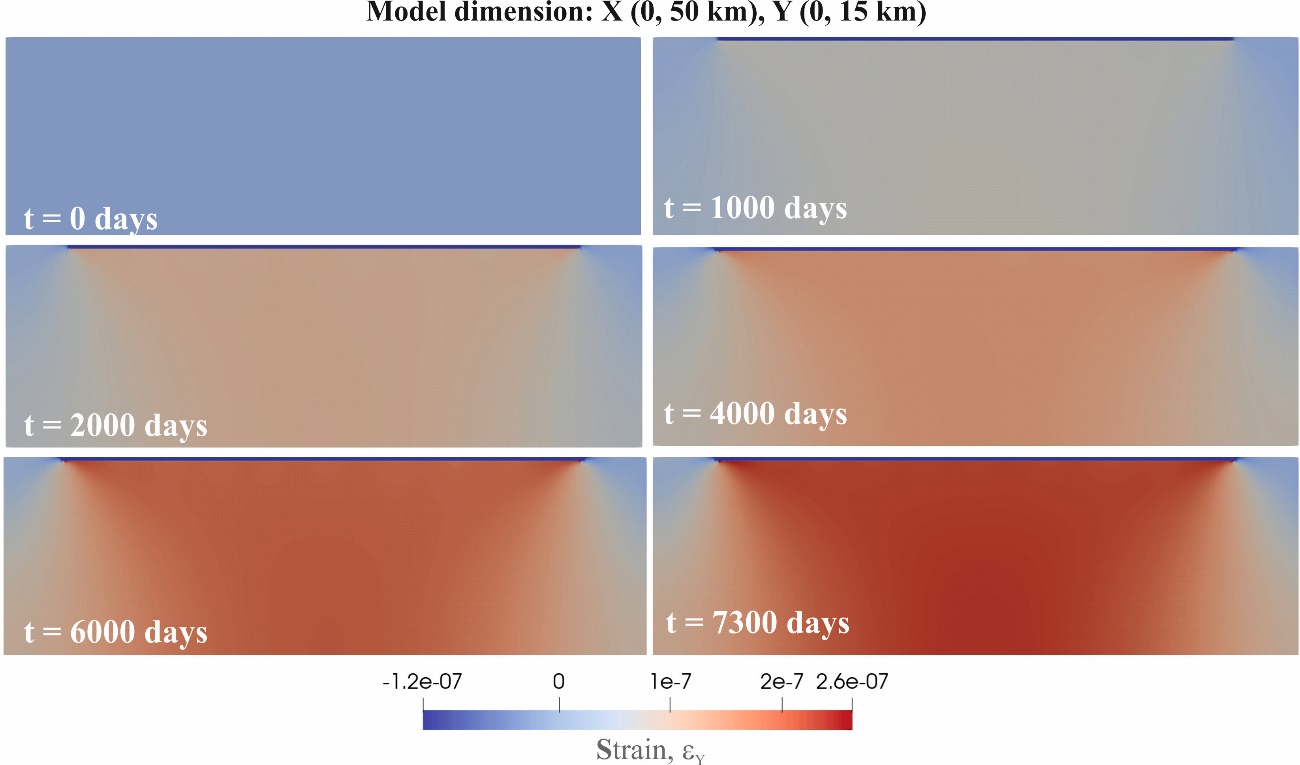


**Figure S6:** Groundwater extraction-induced changes in the $\epsilon_{x}$ and $\epsilon_{y}$ strain components at six different time steps. This figure was generated using ParaView application (version 5.7.0 URL: <https://www.paraview.org/>).


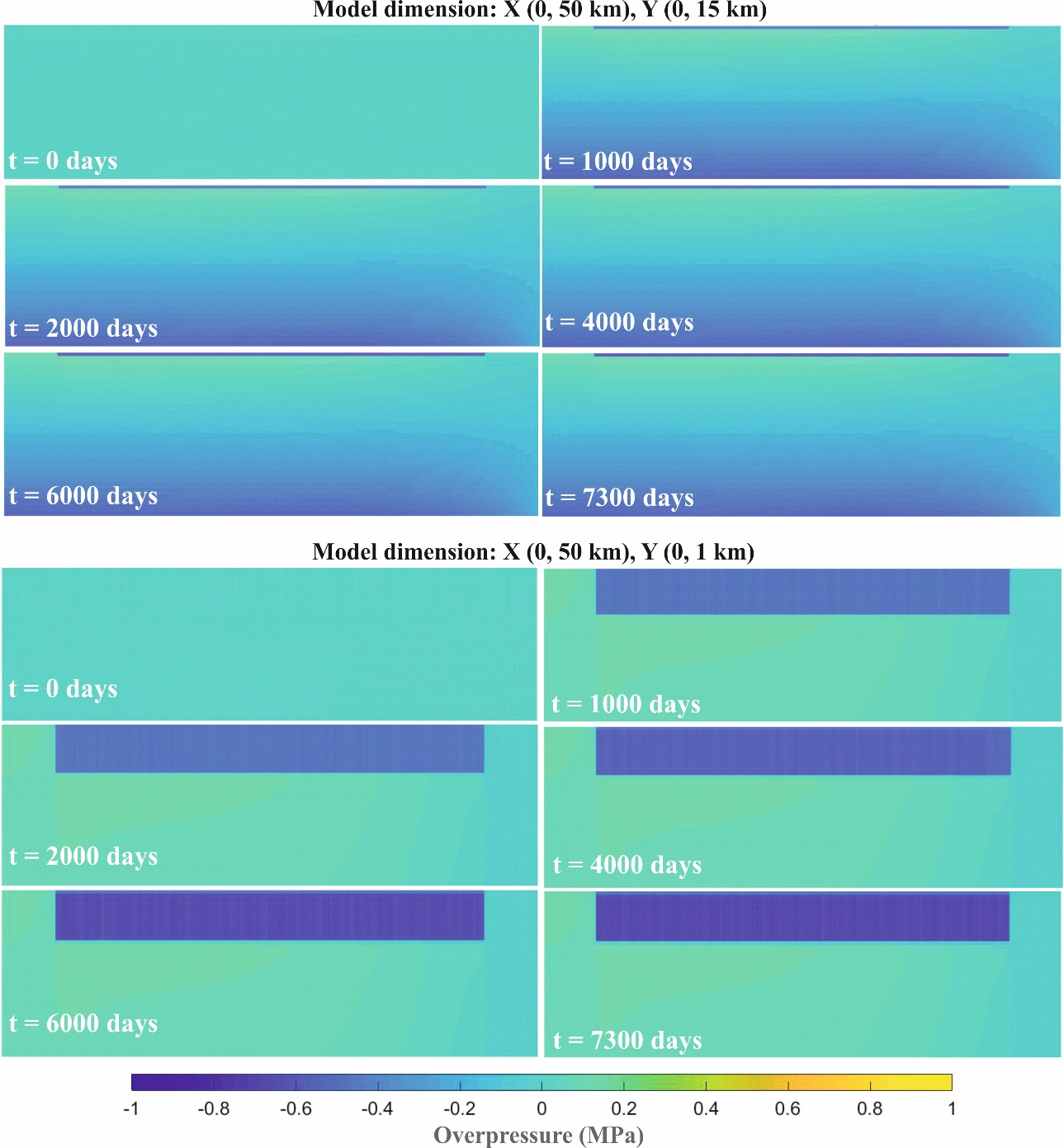


**Figure S7:** Change in pressure within the entire domain (upper panel with Y (0, 15 km)) and within the aquifer (lower panel Y (0, 1 km)) at six different time steps. Groundwater extraction from the aquifer leads to pressure drop within the aquifer. Poroelastic effects lead to pressure drop within basement. This figure was generated using Matlab application (version 18.04a URL: <https://in.mathworks.com/>).

**
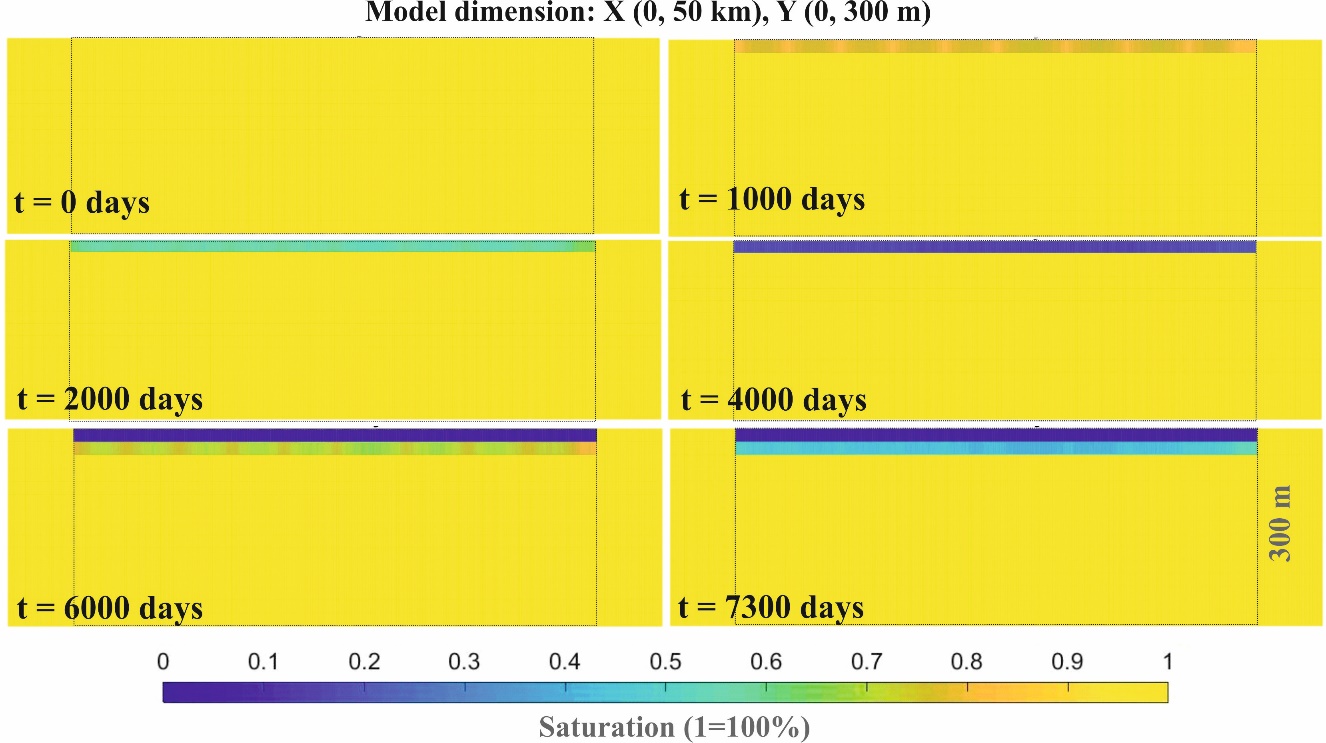
**

**Figure S8:** Groundwater extraction causes a drop in the water saturation within the aquifer (top 300 m of the model) as the water table drops and air substitutes water as the pore fluid. Six time steps are shown to capture the temporal evolution of the saturation field. This figure was generated using Matlab application (version 18.04a URL: <https://in.mathworks.com/>).


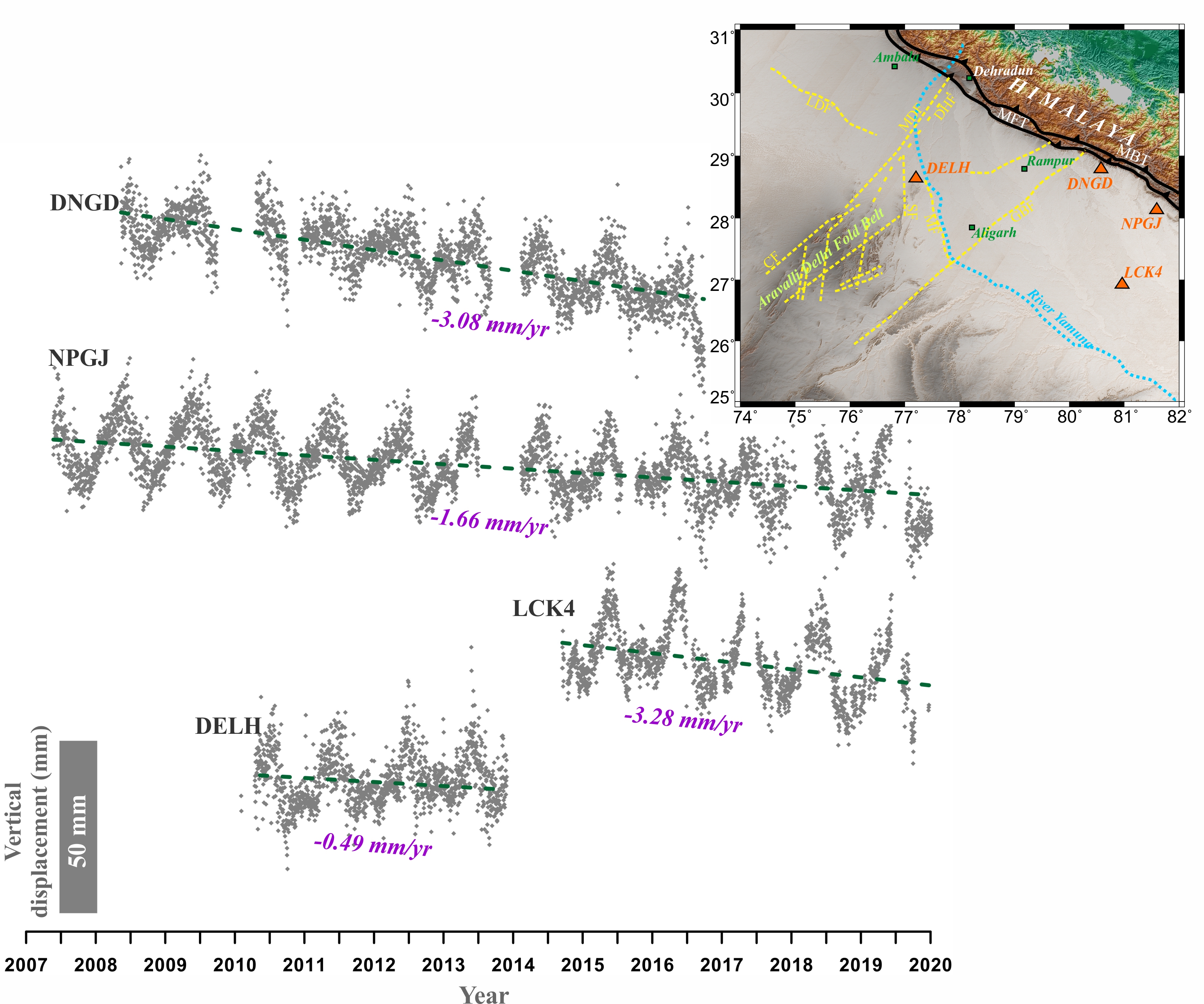


**Figure S9:** Representative GPS vertical time series from Delhi and surrounding regions (marked in Inset map). Note that the vertical displacements show surface subsidence (indicated by negative trends) due to groundwater extraction. This figure was generated using Corel Draw graphical application (version 18 URL: <https://www.coreldraw.com/en>).
